# Systemic inflammation, growth factors, and linear growth in the setting of infection and malnutrition

**DOI:** 10.1016/j.nut.2016.06.013

**Published:** 2017-01

**Authors:** Mark D. DeBoer, Rebecca J. Scharf, Alvaro M. Leite, Alessandra Férrer, Alexandre Havt, Relana Pinkerton, Aldo A. Lima, Richard L. Guerrant

**Affiliations:** a Pediatric Endocrinology, Department of Pediatrics, University of Virginia, Charlottesville, VA; b Developmental Pediatrics, Department of Pediatrics, University of Virginia, Charlottesville, VA; c Institute of Biomedicine, Federal University of Ceará, Fortaleza, Brazil; d Center for Global Health, University of Virginia, Charlottesville, VA

**Keywords:** Nutrition, Diarrhea, Growth, Stunting, IGF-1

## Abstract

**Objectives:**

Deficits in weight gain and linear growth are seen frequently among children in areas where malnutrition and recurrent infections are common. Although both inflammation and malnutrition can result in growth hormone (GH) resistance, the interrelationships of infection, inflammation, and growth deficits in developing areas remain unclear. The aim of this study was to evaluate relationships between low levels of systemic inflammation, growth factors, and anthropometry in a case–control cohort of underweight and normal weight children in northern Brazil.

**Methods:**

We evaluated data from 147 children ages 6 to 24 mo evaluated in the MAL-ED (Interactions of Malnutrition and Enteric Disease) case–control study following recruitment from a nutrition clinic for impoverished families in Fortaleza, Brazil. We used nonparametric tests and linear regression to evaluate relationships between current symptoms of infections (assessed by questionnaire), systemic inflammation (assessed by high-sensitivity C-reactive protein [hsCRP]), the GH insulin-like growth factor-1 (IGF-1) axis, and measures of anthropometry. All models were adjusted for age and sex.

**Results:**

Children with recent symptoms of diarrhea, cough, and fever (compared with those without symptoms) had higher hsCRP levels; those with recent diarrhea and fever also had lower IGF-1 and higher GH levels. Stool myeloperoxidase was positively associated with serum hsCRP. hsCRP was in turn positively associated with GH and negatively associated with IGF-1 and IGF-binding protein-3 (IGFBP-3), suggesting a state of GH resistance. After adjustment for hsCRP, IGF-1 and IGFBP-3 were positively and GH was negatively associated with *Z* scores for height and weight.

**Conclusions:**

Infection and inflammation were linked to evidence of GH resistance, whereas levels of GH, IGF-1, and IGFBP-3 were associated with growth indices independent of hsCRP. These data implicate complex interrelationships between infection, nutritional status, GH axis, and linear growth in children from a developing area.

## Highlights

• Among children in this developing area, symptoms of illness correlated with high-sensitivity C-reactive protein (hsCRP).
• hsCRP correlated positively with growth hormone (GH) and negatively with insulin-like growth factor (IGF)-1 and IGF-binding protein-3 (IGFBP-3).
• IGF-1 and IGFBP-3 correlated positively with height.
• These data are consistent with a model of inflammation-induced GH resistance.

## Introduction

Recurrent infections are associated with lower growth velocity and stunting in children in developing areas of the world [1]. This is supported by previous findings that demonstrated temporal links between slowed growth and repeated infections [2], as well as by more recent cohorts evaluated for growth related to clinically relevant diarrheal [3], [4], [5] and respiratory [6], [7] infections. Enteric infections involving poor growth have been linked to subclinical infections and may exhibit both systemic inflammation and elevations in stool inflammatory markers such as myeloperoxidase, α1 antitrypsin, and neopterin without overt diarrheal symptoms [8]. The presence of systemic inflammation during infection may play a role in growth suppression, as similar linear growth deficits have been noted in other conditions with high levels of systemic inflammation, such as Crohn's disease and juvenile idiopathic arthritis, in which poor growth is associated with high levels of inflammatory markers, low levels of important growth factors such as insulin-like growth factor (IGF)-1, and poor responsiveness of the growth plate [9], [10], [11], [12].

**Fig. 1:**
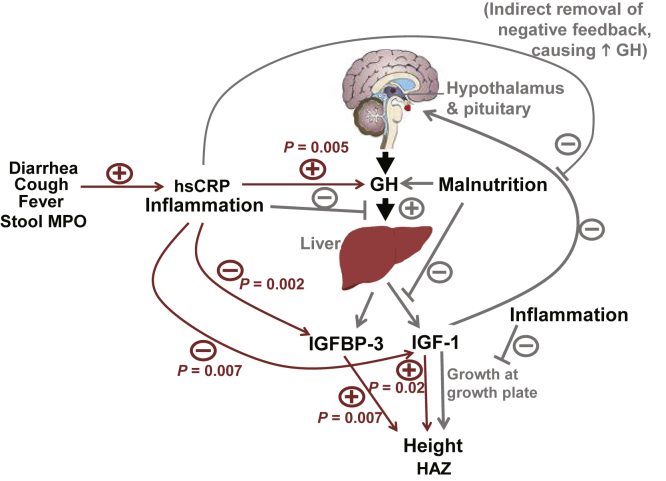
Conceptual model of the effect of inflammation on regulation of the GH-IGF-1 axis. Lines in gray portray associations from preclinical studies and lines in red portray findings from this study. Stimulatory effects or positive associations are indicted (+); inhibition or inverse associations are indicated by (−) signs. Preclinical studies (gray lines) demonstrate effects of inflammation to induce GH resistance at the level of the liver, resulting in decreased expression of IGF-1 and IGFBP-3; this results in an increase in circulating GH by loss of negative feedback of IGF-1 on GH release. IGF-1 is the key mediator of the action of the GH axis on linear growth. The analysis from the present study's cohort found multiple correlations (red arrows, with *P*-values) consistent with these concepts. hsCRP was higher among children with symptoms of illness and positively associated with stool MPO As expected from the model, hsCRP was positively associated with GH, and negatively associated with IGF-1 and IGFBP-3. IGF-1 and IGFBP-3 were in turn positively associated with HAZ, independent of hsCRP levels. GH, growth hormone; HAZ, height-for-age *Z* score; hsCRP, high-sensitivity C-reactive protein; IGF, insulin-like growth factor; IGFBP, IGF binding protein; MPO, myeloperoxidase.

Preclinical models of inflammatory disease have demonstrated direct relationships between systemic inflammation, growth hormone (GH) signaling, and linear growth [13]. Higher systemic inflammation is related to GH resistance at the level of the liver as evidenced by the following:

• higher systemic levels of growth hormone [14],
• lower hepatic production of IGF-1 and IGF binding-protein-3 (IGFBP-3),
• lower systemic levels of IGF-1 and IGFBP-3, and
• slowed linear growth (gray lines in Fig. 1) [15].

Blocking systemic inflammation with antibodies against tumor necrosis factor-α reversed each of these GH signaling outcomes, suggesting direct relationships [15]. Links between infections, inflammatory markers such as C-reactive protein (CRP), and levels of IGF-1 recently have been demonstrated in a cohort of young children followed in Zimbabwe [10]. Similarly, a group of researchers in Uganda studied children presenting with severe acute malnutrition (mean weight-for-height *Z* scores of −4.2), reporting high levels of systemic inflammation and GH and low levels of IGF-1 [16]. However, the degree of inflammation required for suppression of GH signaling is unclear. Although major infections can involve elevations of CRP to >300 mg/L [17], more common infections have been associated with minor elevations in CRP, as tested in the more sensitive assay “high-sensitivity” CRP (hsCRP), producing levels of 15 to 30 mg/L [18], [19].

Thus, the relationship between lower levels of the systemic inflammation and GH signaling in humans requires further investigation. The aim of this study was to evaluate relationships between low levels of systemic inflammation, growth factors, and anthropometry in a case–control cohort of underweight and normal weight children in northern Brazil. Our hypothesis was that even low levels of inflammation would be associated with GH resistance, including lower levels of IGF-1 and IGFBP-3 and higher levels of GH through lack of feedback. A link between inflammation and the GH–IGF-1 axis may have implications for growth faltering among children in developing areas of the world.

## Methods

The present study was performed as an extension of the MAL-ED (Interactions of Malnutrition and Enteric Disease) study with an extended biomarker study, both funded by the Bill and Melinda Gates Foundation. As part of this overall project, a case–control study examining biomarkers of malnutrition and intestinal infection was conducted. The present study represents an ancillary trial of this biomarkers initiative.

The MAL-ED case–control study protocol and consent forms were approved by the local institutional review board (IRB) at Universidade Federal do Ceará, the national IRB, Conselho Nacional de Ética em Pesquisa, Brasília, DF, Brazil, and the IRB at the University of Virginia, VA. Between August 2010 and September 2012, children were recruited for the case–control study at Promotion of Nutrition and Human Development located in Fortaleza, Brazil. Further details including the geographic location, population, demographic characteristics, environmental, and socioeconomic status have been described elsewhere [20]. Malnourished or “case” children were defined as having weight-for-age (WAZ) scores <−2 and matched “nonmalnourished controls” were defined as having a WAZ >−1. Children who required prolonged hospitalization or had serious health issues, such as HIV, tuberculosis, neonatal disease, kidney disease, chronic heart failure, liver disease, cystic fibrosis, congenital conditions, or enteropathy (e.g., Crohn's disease, celiac disease, ulcerative colitis, or malabsorption disease), diagnosed by a physician; or those with a parent or primary caregiver with cognitive deficits or who was <16 y of age were excluded. For mothers who were ages 16 to 17 y, permission of their guardian or the child's father was required for enrollment in the case–control study. Of 484 children screened, 82 declined or failed to meet enrollment criteria. After obtaining informed consent from the responsible parent or guardian, 402 children ages 6 to 26 mo were enrolled (201 cases and 201 controls). Of these, 321 provided fecal samples within 1 mo of enrollment and 292 provided initial blood plasma specimens. Anthropometry was assessed at study visit 1. Age and anthropometric measures were used to generate *Z* scores for length, weight, and weight-for-length according to the World Health Organization growth curves; height-for-age *Z* scores were referred to as HAZ by convention [21]. Stunting was defined as HAZ <−2.

Cases in which blood draw or stool collection were postponed for >1 mo were excluded. Additionally, for all analyses regarding interrelationships between variable categories (anthropometry, survey response, serum measures, and stool measures), only variables obtained <7 d from each other were included.

For the current analysis, cases and controls were compared for variables of interest. With the exception of differences in anthropometry (which was true by design), there were no differences between cases and controls with respect to illness symptoms, hormones, or markers of inflammation. Thus, both groups were combined into a single cohort for analysis.

Blood was spun down and frozen at −80° before transport to the Ligand Core Laboratory of the University of Virginia. The following assays were assessed using the Immulite 2000, with intra- and interassay coefficients of variation (CVs) in parentheses: hsCRP (4.2%, 7.4%), IGF-1 (2.3%, 3.8%), IGFBP-3 (2.6%, 4.3%), GH (2.5%, 2.8%), free T4 (2.9%, 4%), and thyroid-stimulating hormone (TSH: 3.6%, 4.7%).

Fecal biomarkers were included using commercially available enzyme-linked immunosorbent assay kits for myeloperoxidase (MPO; Alpco, Salem, NH, USA), as a marker of neutrophil activity in the intestinal mucosa; α-1 antitrypsin (A1 AT; Biovendor, Candler, NC, USA) to indicate protein loss and intestinal permeability; and neopterin (GenWay Biotech, San Diego, CA, USA) to indicate T-helper cell 1 activity. Each of these was quantified with standard curves run as recommended, and with results being expressed per mg of stool. The intra- and interassay CVs for these assays are: MPO (4.7, 9.7), A1 AT (4.5–6.3, 5–8.2), and neopterin (4.3–11.7, 8.8–13.8).

### Statistical analysis

All statistical tests were performed using SAS 9.4. Measures were assessed for normality. In the case of participant characteristics, non-normally distributed comparisons between groups (e.g., stunted versus nonstunted) were performed using Wilcoxon Rank Sum test. For laboratory variables, the non-normally distributed values were natural-log transformed before further analysis; values were then back-transformed for reporting in tables. We used linear regression to assess relationships between different laboratory variables (e.g., hsCRP and GH axis) and between laboratory variables and anthropometry *Z* scores. All models included adjustment for age and sex. Significance was considered for *P* < 0.05.

## Results

### Participant characteristics

Participant characteristics are shown in Table 1 by stunting and wasting status. By study design, there was a high prevalence of children with stunting in the cohort (58%); there was a lower proportion of children with wasting (16%). Children with either stunting or wasting were significantly older than unaffected children (16.3 versus 10.6 mo for stunting versus nonstunting and 17.5 versus 13.7 mo for wasting versus nonwasting; both *P* < 0.01). Children with stunting had lower birthweights compared with those without stunting. For stunting status, serum and stool measures only differed with respect to IGFBP-3 levels, which were lower in stunted than in nonstunted participants (2.22 versus 2.63 mg/L; *P* = 0.034). None of the laboratory measures differed by wasting status. Because of the relationship between age and growth factor levels, we performed linear regression for the relationship between stunting status and wasting status (separately), adjusting for age. There remained no significant difference between groups (data not shown).

### Illness symptoms and serum measures

Table 2 displays the levels of hsCRP, IGF-1, and GH by the presence of illness symptoms. There was a relatively high proportion of children who over the previous week had experienced diarrhea (12%), cough (39%), and/or fever (34%). Levels of hsCRP were higher among children who had experienced diarrhea or cough over the previous day or week (all *P* < 0.01), whereas with respect to fever, hsCRP levels were only higher among those with overt diarrhea over the previous week (*P* = 0.017). Levels of IGF-1 were lower and GH levels were higher, among children with diarrhea over the previous week (both *P* < 0.05) and those with fever over the previous day (both *P* < 0.0001). Levels of IGF-BP3 were similar between children with and without illness symptoms (data not shown).

### Correlates of hsCRP with hormones and stool measures

We next evaluated for the associations between interactions between hsCRP and the GH axis by considering GH, IGF-1, and IGFBP-3 as the outcomes and hsCRP as the independent variable (Table 3). hsCRP was positively associated with levels of GH (*P* = 0.005) and negatively associated with IGF-1 (*P* = 0.002) and IGFBP-3 (*P* = 0.008). hsCRP was not related to concurrent levels of free T4 or TSH.

In evaluating for potential connections between stool markers of inflammation and serum markers of systemic inflammation, we assessed hsCRP as the outcome and stool measures as the independent variables. Stool MPO was positively associated with serum hsCRP (*P* = 0.002).

### Correlates of anthropometric measures

Finally, we assessed for relationships between serum and stool factors and growth indices (Table 4). hsCRP was not significantly linked to anthropometric measures. GH levels were negatively associated and IGFBP-3 levels were positively associated with HAZ, WAZ, and weight-for-height (WHZ; all *P* < 0.05); IGF-1 levels exhibited a trend toward positive association with HAZ and WAZ (both *P* = 0.07). Following further adjustment for current hsCRP, GH was negatively associated (*P* < 0.01) and IGF-1 (*P* < 0.05) and IGFBP-3 (*P* < 0.01) were positively associated with HAZ and WAZ, whereas GH and IGFBP-3 were additionally associated with WHZ. Free T4, TSH, and stool measures were not significantly linked to anthropometric measures.

## Discussion

In a cohort of significant poverty from northeast Brazil, we noted associations between a mild degree of systemic inflammation as marked by higher levels of hsCRP and both higher levels of GH and lower levels of IGF-1 and IGFBP-3. In turn, higher GH and lower IGFBP-3 levels were associated with shorter stature. These findings are consistent with a model of inflammation driving hepatic resistance to GH signaling as noted in preclinical studies (Fig. 1) [15]. To our knowledge, this is the first study from an area with poverty and likely endemic infections demonstrating both associations between inflammation and the GH axis and between the GH axis and anthropometry in the same cohort. Although preliminary, these data may have implications regarding downstream effects of systemic inflammation and malnutrition in early childhood.

hsCRP elevations have been noted during minor acute infections [18], [19], as was noted in this study among children with recent symptoms of illness. However, these symptomatic children comprised the minority of the overall cohort. Nevertheless, only among children with recent diarrhea had both hsCRP levels higher and IGF-1 levels lower. Other sources of hsCRP elevation beyond current infections in this cohort remain unclear.

In this study, we did not find a relationship between levels of hsCRP and anthropometric measures themselves, but only to growth factors. This underscores the complexity of these relationships in that although current hsCRP is related to levels of growth factors, current levels of GH, IGF, and IGFBP-3 levels were related to height and weight independent of hsCRP levels, suggesting that there are other important biological influences on these processes as well. Linear growth is clearly a long-term process and current elevations in hsCRP may not indicate the long-standing inflammation that would be expected to be required for clinically evident growth deficits. Additionally, noninflammatory factors are important in growth factor production, including genetics related to GH and its signaling. Thus, further information is needed to elucidate the extent to which infections and inflammation are responsible for any lower height in the children of this cohort. Furthermore, social factors may also be important for both exposure to infections and poor nutrition—which may contribute to lower growth in this setting.

In addition to influencing growth factor production, systemic inflammation can affect the responsiveness of the growth plates themselves [13]. This is seen in preclinical studies demonstrating suppressive actions of inflammatory cytokines on chondrocytes corresponding to the length of exposure [12]. Malnutrition is likely to play a role in growth plate responsiveness through factors such as fibroblast growth factor (FGF)21, which is elevated in fasting and directly inhibits GH binding to chondrocytes [22]; overexpression of FGF21 in mouse models results in poor linear growth [23]. However, remaining issues regarding differential effects of long-term malnutrition and inflammation on FGF21 and growth plate response require further investigation [12]. Unfortunately, we were limited in serum supply and unable to test FGF21 levels in these participants.

Some of the difficulties in separating the potential effects of systemic inflammation and malnutrition on GH resistance rest in that many cases of systemic inflammation occur alongside malnutrition, including Crohn's disease and subacute enteric disease [1], [11]. Cases of malnutrition in resource-poor areas of the world frequently are associated with higher levels of systemic inflammation as well, suggesting the potential for current infections fueling poor weight gain [1], [8], [16], [24]. Because GH is a counter-regulatory hormone that mobilizes energy stores during malnutrition, elevations in GH such as we observed can be seen in states of undernutrition [16], [24], [25]. Under normal conditions, IGF-1 and IGFBP-3 are produced by the liver in response to GH. However, during states of pure malnutrition (such as in anorexia nervosa), IGF-1 levels are low, demonstrating GH resistance [25], [26]. Although IGF-1 is commonly seen as a marker of nutritional status, IGFBP-3 is more stable in states of under nutrition and is more commonly seen as an indicator of adequate GH signaling [27]. That hsCRP was associated with higher GH levels and lower levels of IGF-1 and IGFBP-3 (suggesting lower hepatic response to GH) suggests against poor nutrition as the only reason for these associations, particularly given the lower levels of IGFBP-3 [27].

We found higher levels of hsCRP in children with current or recent symptoms of infection, including diarrhea, cough, and fever. This has been noted in previous studies and is likely linked to the immune response to underlying viral or bacterial infection [28]. With the exception of those with current fever (who had lower HAZ, WAZ, and WHZ), children with symptoms of infection did not have differences in anthropometry, potentially underscoring that these episodes do not necessarily indicate a pattern of recurring illnesses over time. However, as noted previously, repeated bouts of illness may lead to more prolonged exposure to systemic inflammation and are associated with faltering growth [2].

This study had multiple limitations. The original case–control study defined malnutrition using WAZ instead of WHZ, likely a more appropriate way to classify poor nutritional status. We only analyzed cross-sectional measure of height instead of assessments of linear growth over time. We did not directly assess for respiratory pathogens among participants and were thus unable to test for links between current infections and inflammation and growth factor production. We had hsCRP as the only marker of systemic inflammation and lacked serum levels of inflammatory cytokines. We assessed random GH levels instead of the more valid method of assessing stimulated levels; nevertheless random levels are on average higher in states of GH resistance when considered for a full cohort [16], [24], [25]. We were unable to measure GH-binding protein as a further assessment of GH regulation. We also lacked more sophisticated markers of nutritional status, such as prealbumin (as a marker of mild nutritional deficits), calorie counts, and direct measurement of fat mass such as dual-energy x-ray absorptiometry analysis. However, the study had strengths, including assessment of multiple aspects of infection, systemic inflammation, growth factors, and anthropometry in a single cohort.

## Conclusions

We found both connections between current inflammation and GH axis levels (with inverse relationships between hsCRP and IGF-1 and IGFBP-3) and between current growth factors and HAZ and WAZ. These data are consistent with models of infection and inflammation suppressing growth factor expression and ultimately suppressing growth. Further research is needed to assess long-term relative effects of suppressing inflammation and improving nutritional status on improving growth outcomes, particularly in developing areas with high rates of linear growth failure.

## Figures and Tables

**Table 1:** Participant characteristics for anthropometric and hormone results by stunting status

Characteristic, mean (SD) unless otherwise noted	Overall	Stunted (n = 86)	Nonstunted (n = 62)	*P* value∗	Low weight-for-length (<2 SD) (n = 23)	Normal weight-for-length (n = 125)	*P* value∗
Maternal data							
Age (y), median (IQR)	25 (22–32)	25 (20–32)	25.5 (22–31)	0.875	24.5 (22–31)	25.0 (22–32)	0.596
Income ($), median (IQR)	373 (300–540)	390 (270–600)	350 (300–481)	0.556	650 (500–1500)	622 (500–890)	0.596
Education (y), median (IQR)	8 (6–11)	9 (6–11)	8 (5–11)	0.824	8 (6–12)	8.5 (6–11)	0.657
Height, cm	151.8 (6.4)	150.3 (6.6)	153.9 (5.5)	0.001	148 (144–151.5)	152 (148–156.5)	0.014
Weight, kg	57.3 (12.5)	55.8 (11.9)	59.3 (13)	0.082	53.5 (50–58.1)	57.0 (47.1–66.5)	0.288
BMI, kg/m^2^	24.9 (5.3)	24.8 (5.2)	25.1 (5.5)	0.636	24.8 (23.1–28.3)	24.1 (20.4–28.3)	0.912
Child data							
Age (mo), median (IQR)	13.5 (9.1–19.1)	16.3 (11.7–19.9)	10.6 (7.3–16.4)	<0.001	17.5 (5.4)	13.7 (5.6)	0.004
% female	48.3	46.5	50.8	0.607	34.8	50.8	0.148
Birthweight, kg	2.89	2.57	3.31	<0.0001	2.67	2.93	0.106
WAZ	−1.31 (1.58)	−2.39 (0.95)	0.17 (1.18)	<0.0001	−3.28 (0.63)	−0.95 (1.51)	<0.0001
HAZ	−1.76 (1.38)	−2.89 (0.75)	−0.21 (0.83)	<0.0001	−2.82 (1.00)	−1.56 (1.55)	<0.0001
WHZ	−0.52 (1.46)	−1.20 (1.16)	0.39 (1.26)	<0.0001	−2.58 (0.37)	−0.15 (1.22)	<0.0001
Serum measures							
GH (ng/mL), median IQR	1.71 (0.81–3.05)	1.8 (1.2–3.5)	1.6 (0.7–2.4)	0.075†	1.73 (1.26–2.18)	1.69 (0.81–3.21)	0.976†
IGF-1 (ng/mL), median IQR	33.4 (24.9–66.7)	32.0 (24.9–68.8)	34 (24.9–60)	0.775†	33.3 (24.9–58.5)	33.5 (24.9–67.1)	0.616†
IGFBP-3, mg/L	2.40 (0.94)	2.22 (0.99)	2.63 (0.82)	**0.034**	2.07 (1.06)	2.47 (0.90)	0.174
Free T4, μg/dL	1.09 (0.18)	1.11 (0.17)	1.08 (0.18)	0.428	1.09 (0.20)	1.09 (0.17)	0.897
TSH, μU/mL	3.18 (1.88)	3.01 (1.76)	3.43 (2.03)	0.210	2.63 (1.65)	3.28 (1.90)	0.104
hsCRP (mg/L), median IQR	1.12 (0.37–4.91)	1.09 (0.30–5.03)	1.13 (0.42–4.18)	0.916†	0.51 (0.20–5.42)	1.13 (0.38–4.41)	0.504
Stool measures							
Neopterin, median IQR	1335 (746–2174)	1168 (630–1961)	1793 (821–2595)	0.420†	841 (298–1816)	998 (518–2044)	0.276†
α-1 antitrypsin, median IQR	10.4 (4.3–21.1)	10.2 (3.9–21.9)	10.7 (6.4–20.3)	0.546†	10.3 (4.4–21.8)	10.9 (4.6–19.7)	0.653†
Myeloperoxidase, median IQR	3439 (1478–6954)	3114 (1602–6606)	3881 (1388–7085)	0.322†	2427 (1759–5647)	3481 (1474–7267)	0.925†
Illness measures							
Fever previous wk, %	35.6	34.9	36.5	0.838	47.8	32.3	0.182
Cough previous wk, %	20.1	24.4	14.3	0.128	12.1	19.1	0.439
Diarrhea previous wk, %	12.8	14	11.1	0.607	8.7	13.5	0.526

BMI, body mass index; hsCRP, high-sensitivity C-reactive protein; GH, growth hormone; HAZ, height-for-age *Z* score; IGF, insulin-like growth factor; IGFBP, IGF binding protein; IQR, interquartile range; TSH, thyroid-stimulating hormone; WAZ, weight-for-age *Z* score; WHZ, weight-for-height *Z* score

*P* value <0.05 is indicated in bold

∗ Demographic variables that were skewed were compared using Wilcoxon Rank Sum.

† *P* value for comparison of log-transformed values.

**Table 2:** hsCRP, IGF-1, and GH levels in children by recent/current symptoms of infection

Illness symptom	hsCRP				IGF-1				GH			
	n	Affected children	Unaffected children	*P* value∗	n	Affected children	Unaffected children	*P* value∗	n	Affected children	Unaffected children	*P* value∗
		hsCRP†, mean (SD)	hsCRP†, mean (SD)			IGF-1†, mean (SD)	IGF-1†, mean (SD)			GH†, mean (SD)	GH†, mean (SD)	
Diarrhea previous d	3/131	12.7 (1.63)	1.39 (4.90)	**0.006**	3/131	30 (1.4)	42.1 (1.8)	0.199	2/106	1.53 (1.08)	1.51 (2.76)	0.887
Diarrhea previous wk	16/131	6.62 (4.39)	1.20 (4.26)	**0.003**	16/131	32.1 (1.5)	43.4 (1.8)	**0.017**	13/106	2.43 (2.20)	1.41 (2.77)	**0.038**
Cough previous d	28/131	3.46 (6.69)	1.16 (4.81)	**0.001**	28/131	42.9 (1.9)	42.3 (1.8)	0.781	21/106	1.74 (2.62)	1.46 (2.77)	0.455
Cough previous wk	51/131	2.46 (4.81)	1.06 (4.71)	**0.003**	51/131	40.0 (1.7)	42.9 (1.8)	0.518	39/106	1.79 (2.83)	1.36 (2.67)	0.185
Fever previous d	2/131	3.59 (20.91)	1.45 (4.90)	0.745	2/131	24.8 (1.0)	42.1 (1.8)	**<0.0001**	2/106	2.54 (1.05)	1.49 (2.76)	**<0.0001**
Fever previous 2 wk	45/131	3.36 (5.21)	1.15 (4.66)	**0.017**	45/131	38.9 (1.6)	43.4 (1.9)	0.257	37/106	1.72 (2.53)	1.40 (2.85)	0.302

GH, growth hormone; hsCRP, high-sensitivity C-reactive protein; IGF, insulin-like growth factor

*P* values <0.05 are indicated in bold

∗ *P* value by Satterthwaite, assuming unequal variance.

† Means and comparison between affected and unaffected children were performed using log-transformed values; back-transformed values of these are displayed.

**Table 3:** Linear regression of relationships between hsCRP∗ and growth hormone axis

	β coefficient	Confidence interval	R^2^	*P* value
hsCRP as predictor†				
GH∗	0.158	0.050–0.266	**0.108**	**0.005**
IGF-1∗	−0.091	−0.146 to −0.039	**0.136**	**0.002**
IGFBP-3	−0.162	−0.280 to −0.043	**0.079**	**0.008**
hsCRP as outcome†				
Neopterin∗	0.190	−0.149 to 0.529	0.017	0.294
α-1 antitrypsin∗	0.024	−0.253 to 0.301	0.017	0.865
Myeloperoxidase∗	0.358	0.121–0.595	**0.081**	**0.003**

GH, growth hormone; hsCRP, high-sensitivity C-reactive protein; IGF, insulin-like growth factor; IGFBP, IGF binding protein

*P* values <0.05 are indicated in bold

∗ Log-transformed.

† All models further adjusted for age and sex.

**Table 4:** Linear regression of growth-related hormones with HAZ and WAZ∗

Predictor	HAZ			WAZ			WHZ		
	β coefficient	95% CI	*P* value	β coefficient	95% CI	*P* value	β coefficient	95% CI	*P* value
hsCRP†	0.031	−0.118 to 0.181	0.680	0.062	−0.095 to 0.219	0.436	0.060	−0.076 to 0.120	0.383
GH†	−0.305	−0.565 to −0.046	**0.021**	−0.359	−0.639 to −0.080	**0.012**	−0.275	−0.526 to −0.024	**0.032**
Model with hsCRP	−0.354	−0.622 to −0.087	**0.010**	−0.431	−0.718 to −0.145	**0.004**	−0.336	−0.594 to −0.078	**0.011**
IGF-1†	0.039	−0.028 to 0.817	0.067	0.407	−0.038 to 0.852	0.073	0.292	−0.096 to 0.681	0.139
Model with hsCRP	0.448	0.010–0.886	**0.045**	0.485	0.026–0.945	**0.026**	0.361	−0.039 to 0.762	0.077
IGFBP-3	0.428	0.118–0.738	**0.007**	0.439	0.112–0.767	**0.009**	0.326	0.041–0.611	**0.026**
Model with hsCRP	0.451	0.127–0.776	**0.007**	0.496	0.155–0.836	**0.005**	0.384	0.089–0.679	**0.011**

GH, growth hormone; HAZ, height-for-age *Z* score; hsCRP, high-sensitivity C-reactive protein; IGF, insulin-like growth factor; IGFBP, IGF binding protein; WAZ, weight-for-age *Z* score; WHZ, weight-for-height *Z* score

*P* values <0.05 are indicated in bold

∗ Each linear regression model included adjustment for sex and age. An additional model was analyzed for GH, IGF-1, and IGFBP-3 with further adjustment for hsCRP as shown.

† Log-transformed.
